# Improvement of magnetic resonance imaging using a wireless radiofrequency resonator array

**DOI:** 10.1038/s41598-021-02533-3

**Published:** 2021-11-29

**Authors:** Akbar Alipour, Alan C. Seifert, Bradley N. Delman, Philip M. Robson, Raj Shrivastava, Patrick R. Hof, Gregor Adriany, Zahi A. Fayad, Priti Balchandani

**Affiliations:** 1 BioMedical Engineering and Imaging Institute and Friedman Brain Institute, Icahn School of Medicine at Mount Sinai, 1470 Madison Ave., 1st Floor, New York, NY 10029 USA; 2 Department of Radiology and Friedman Brain Institute, Icahn School of Medicine at Mount Sinai, 1470 Madison Ave., 1st Floor, New York, NY 10029 USA; 3 Department of Neurosurgery and Friedman Brain Institute, Icahn School of Medicine at Mount Sinai, Annenberg 8, One Gustave L Levy Pl, New York, NY 10029 USA; 4 The Nash Family Department of Neuroscience and Friedman Brain Institute, Icahn School of Medicine at Mount Sinai, One Gustave L. Levy Place, Box 1639, New York, NY 10029 USA; 5 Department of Radiology, Center for Magnetic Resonance Research, University of Minnesota, 2021 6th Street SE, Minneapolis, MN 55455 USA

**Keywords:** Anatomy, Medical research, Engineering, Nanoscience and technology, Physics

## Abstract

In recent years, new human magnetic resonance imaging systems operating at static magnetic fields strengths of 7 Tesla or higher have become available, providing better signal sensitivity compared with lower field strengths. However, imaging human-sized objects at such high field strength and associated precession frequencies is limited due to the technical challenges associated with the wavelength effect, which substantially disturb the transmit field uniformity over the human body when conventional coils are used. Here we report a novel passive inductively-coupled radiofrequency resonator array design with a simple structure that works in conjunction with conventional coils and requires only to be tuned to the scanner’s operating frequency. We show that inductive-coupling between the resonator array and the coil improves the transmit efficiency and signal sensitivity in the targeted region. The simple structure, flexibility, and cost-efficiency make the proposed array design an attractive approach for altering the transmit field distribution specially at high field systems, where the wavelength is comparable with the tissue size.

## Introduction

The number of ultra-high-field (UHF) magnetic resonance imaging (MRI) systems with field strengths of 7 Tesla (7T) or higher are distributed significantly by increasing the interest in neuroscientific applications and clinical research^1–5^. In 2017, “Comformité Européenne” mark was given for a 7T MRI system, indicating safety and environmental protection standards^6^, and later the same year, the United States Food and Drug Administration (FDA) approved the first clinical 7T system^7^. UHF magnets have begun to satisfy the growing demand for increased signal-to-noise ratio (SNR), detailed spatial information, and functional contrast^8–11^. Enhanced signal can be used to reduce the scan time while simultaneously improving the spatial resolution necessary to visualize small brain structures in greater detail. To this end, various protocols have exploited the higher SNR to improve visualization of the supratentorial brain and skull base, and have even enhanced visualization of layers within the cerebellum^12–16^. The value of UHF MRI in modern neuroimaging is demonstrated by the ever-increasing number of 7T scanners installed all over the world. Although 7T MRI was not integrated into clinical settings until 2017, there are now more than 87 installations worldwide and this number is expected to increase rapidly in the coming years^17^.

The radiofrequency (RF) MRI coils used to transmit RF energy into the body and receive MR signals from it play a critical role in determining image quality. The uniformity of the transmit field (${B}_{1}^{+}$) generated by the RF coils is a necessary need in MRI applications in terms of SNR and image uniformity. The most commonly commercially available coils specifically at 7T head MRI consist of a relatively short single channel transmit birdcage coil surrounding a 32-channel receive array. These coils are limited in their coverage because of the technical challenges associated with the RF wavelength effect^18, 19^. As the wavelength in the tissue becomes comparable with the body dimensions, the birdcage coils’ ${B}_{1}^{+}$ homogeneity and efficiency becomes insufficient^20, 21^. Locations of increased and decreased transmit efficiency occur at body-specific locations, causing an inhomogeneous flip angle spatial distribution. Consequently, areas of artifactually high and low signal often appeared in the posterior fossa (cerebellum, brainstem, and other caudal areas of the brain, Supplementary Fig. S1). The difficulty of covering the entire brain, including posterior fossa, has led to most 7T applications to focus on specific regions of the brain^22, 23^.

It is possible to ameliorate the ${B}_{1}^{+}$ non-uniformity caused by the RF wavelength effect using active RF shimming techniques. One such technique is parallel transmission (pTx), which uses multiple transmit coils to improve ${B}_{1}^{+}$ homogeneity at UHF MRI systems^24–28^. The results indicated that pTx can significantly enhance ${B}_{1}^{+}$ uniformity across the entire brain. However, pTx systems clinically are limited by the hardware complexity and difficulty ensuring compliance with local specific absorption rate (SAR) safety limitations compared with a single-transmit configuration^29^.

As an alternative to pTx, passive RF shimming methods have been proposed, which have historically been the only clinically feasible way to homogenize ${B}_{1}^{+}$ distribution in a region of interest (ROI). One of these methods is using dielectric pads (DPs; high permittivity material with ${\varepsilon }_{r}$>50)^30, 31^. DPs are widely used in MRI to improve the transmit efficiency and increase SNR^32–34^. The resultant benefits can be explained by the modified Ampeère’s law, displacement currents within the DPs produce a secondary local RF field which augments the applied ${B}_{1}^{+}$ field. However, there are limitations of conventional DPs, which are usually comprised of mixtures of liquids and ceramic powders: their material parameters can change over time, and some ingredients may be bioincompatible. Artificial dielectric was introduced as an alternative to conventional DPs to avoid some of the disadvantages of conventional pads^35^. Artificial dielectrics are typically periodic structures made of metal or dielectric elements that support the propagation of slow waves with similar phase velocities in the operational band as in natural dielectric materials^36^. Simulated phantom results at 7T show that the artificial dielectrics can obtain the same increase in the ${B}_{1}^{+}$ distribution as the conventional DP^35^. Similar approaches have been reported using resonant metal-dielectric metasurfaces^37, 38^, with hybrid structure comprised of a two-dimensional metamaterial surface and a very high permittivity dielectric substrate, which enhance the local performance of MRI coils^39^. One such study at 7T demonstrated the utility of the metasurface acquiring in vivo human brain images and proton MR spectra with enhanced local sensitivity in the visual cortex on a commercial 7T system^40^. However, the metasurface has a complex structure and may inadequately regulate the hyperintense MR signal in its close vicinity, possibly impairing anatomical information near the structure of interest.

Another passive shimming method to locally improve the ${B}_{1}^{+}$ of the birdcage coil is a non-resonant surface RF coil, appropriately coupled to a volume transmit coil^41^. Theoretical analysis and numerical simulation results demonstrated the feasibility and effectiveness of this method in the human head MRI at 7T.

In our previous study, we showed that using an array of 3 receive-only coupled elements can improve the SNR in the target region^42^. In this study, we aim to improve brain MRI at 7T focusing on the posterior fossa using such an RF array placed against the posterior and caudal portion of the head inside a conventional single-channel transmit MRI head coil. We propose a simple and practical passive resonant RF array design, which works in conjunction with conventional transmit/receive (Tx/Rx) MRI coils to improve the transmit efficiency, signal sensitivity, and anatomical coverage of conventional coils. This design consists of an array of geometrically decoupled resonator elements patterned on a flexible substrate. The addition of an inductively coupled RF array enhances the transmit efficiency during the RF excitation and signal sensitivity during the signal reception near the target region. The tunable and size-adjustable structure of the passive RF resonator array allow to be easily implemented in MRI applications to improve the performance in terms of visualizing regions with reduced transmit efficiency and signal sensitivity, specifically at UHF systems. This concept is generalizable to different field strengths and anatomy of interest.

## Results

### RF array electromagnetic simulations

**Figure 1:**
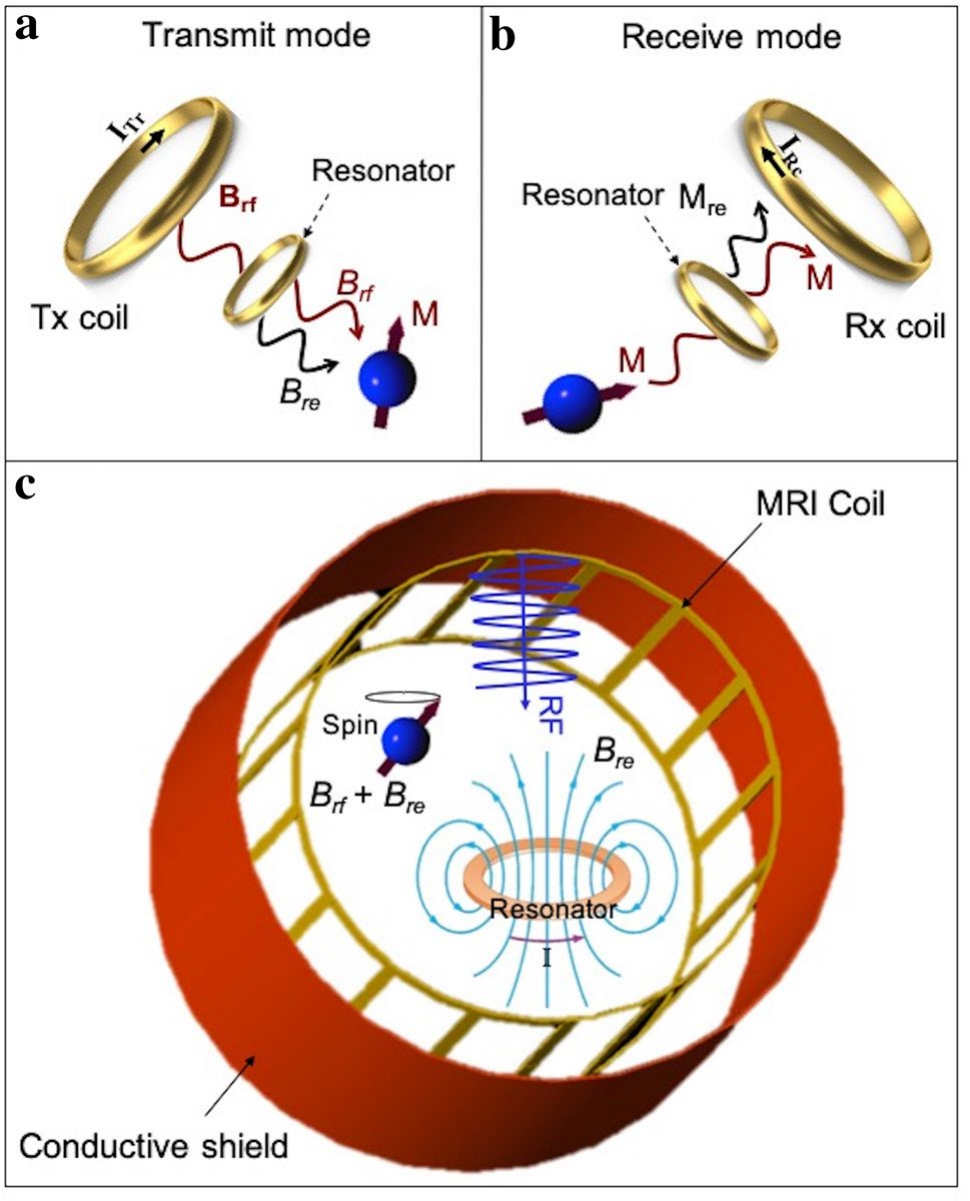
Illustration of inductive coupling between the Tx/Rx MRI coil and an RF passive resonator. (**a**) The resonator locally amplifies transmit magnetic field (${B}_{rf}$) generated by the Tx coil and results in an additional magnetic field (${B}_{re}$). Therefore, spins (symbolized by a blue sphere) experience larger excitation field, which resulted in larger transverse magnetization (M). (**b**) During the receive phase, the resonator couples with the M and Rx coil and enhances the MR signal induced on the Rx coil. (**c**) A schematic of a Tx/Rx birdcage MRI coil which is inductively coupled with a passive RF resonator. The interaction of the RF excitation and the resonator results in a secondary magnetic field, ${B}_{re}$ that locally enhances the transmit efficiency.

The fundamental principle behind the proposed technique is that inductive coupling between the passive RF resonator and MRI Tx/Rx coils results in ${B}_{1}^{+}$ and receive magnetic field (${B}_{1}^{-}$) enhancement. This principle originates from (i) inductive coupling between the resonator and RF excitation that leads to increase in the transmit efficiency (Fig. 1a); and (ii) inductive coupling between the resonator and magnetization vector ($M$) during the reception that leads to receive-signal amplification (Fig. 1b). The transmit magnetic field, ${B}_{rf}$ of the transmit coil inductively couples to the RF resonator, the reaction of the resonator with excitation field leads to circulating current in the resonator, which results in a local magnetic field, ${B}_{re}$ as illustrated in Fig. 1c. ${B}_{re}$ is an additional field provided by the resonator inductance that is added to the original excitation field and enhancing the RF excitation over the object^43, 44^.

**Figure 2:**
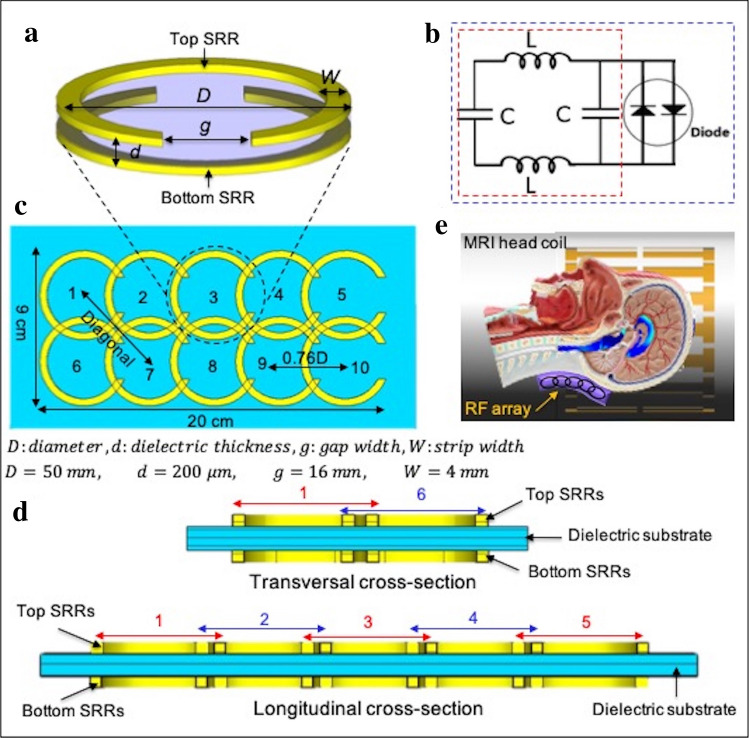
The modeling of the RF array. (**a**) 3D schematic of a passive resonator (BCSRR), which consists of two counter-oriented SRRs. BCSRR design parameters also are shown in 3D model, ***D*** is the diameter, ***W*** is the strip width, ***d*** is the dielectric thickness, and ***g*** is the gap width. In our design, we chose ***D*** = 50 mm***, d*** = 200 μm, ***g*** = 16 mm, and ***W*** = 3 mm. (**b**) Electrical circuit model of the tuned BCSRR (encircled with red dashed). C is the built-in distributed capacitance and L is the inductance. The resistance (R) is not shown in this model. (**c**) A schematic of a 10-element RF resonator array consisting of 10 resonators in a 2 × 5 matrix. An anti-parallel cross diode is used to detune the strongly coupled elements from RF excitation to avoid RF over-flipping (diode is not shown in the 3D model). The corresponding circuit model is encircled with blue dashed in (**c**). (**d**) Cross-sectional view of the array in both transverse and longitudinal axis. The array consists of three layers; two top and bottom metal layers and a dielectric layer sandwiched in between. A thin layer of an insulator is implemented to avoid the physical contact between the overlapping elements (not shown here). (**e**) Schematic of the MRI head coil, wireless RF array, and human model. The array is positioned at the base of the skull such that extends outside from the coil to extend the spatial coverage.

A single RF resonator was modeled as a circular broadside-coupled split-ring-resonator (BCSRR)^45^, which is a 3-layer structure consisting of a flexible dielectric substrate (${\varepsilon }_{r}$=3.4) sandwiched between two split-ring resonators (SRRs), where SRRs are counter-oriented (180° rotated relative to each other; Fig. 2a). The SRR is an enclosed metal loop with a gap $(g)$ along the loop. An equivalent circuit model of a BCSRR is shown in Fig. 2b encircled with a red dashed line, where the resonator is modeled as a series RLC circuit with a resistance (R), distributed capacitance (C), and inductance (L). Electrical characteristics of the RF resonator [resonance frequency (${f}_{0}$) and Q-factor] rely on C, L, and R, which depend on four design parameters: (i) diameter ***D***, (ii) dielectric thickness ***d***, (iii) gap width $g$, and (iv) strip width ***W***.

A 10-element wireless passive RF resonator array was designed using 10 BCSRRs. The elements were oriented as a 2 × 5 matrix (Fig. 2c). The array is a multilayer laminated structure consisting of two metal layers and a dielectric layer as shown in the transverse and longitudinal cross-sectional planes (Fig. 2d). To avoid the physical contact due to overlapping elements on the same plane a thin layer of an insulator is implemented (not shown in the Fig. 2d). The critical overlap technique was used to decouple adjacent elements^46^. As was illustrated in Fig. 2c, $y$ is the center-to-center distance between two inline neighbor elements. The mutual coupling between these elements is minimized when $y=0.76D$. Simulated S-parameters show a transmission coefficient (${S}_{21}$) of − 22 dB and − 18 dB at 312 MHz for the geometrically decoupled inline element pairs and diagonal elements, respectively.

To obtain optimized transmit efficiency in the presence of the resonator the off-resonance frequency of the resonators was adjusted 5% above the Larmor frequency (further electromagnetic (EM) formulations are provided in Supplementary Information B).

The array was placed at the posterior position, between the head and the coil: 2 cm away from the coil and 0.3 cm away from the model illustrated in Fig. 2e.

**Figure 3:**
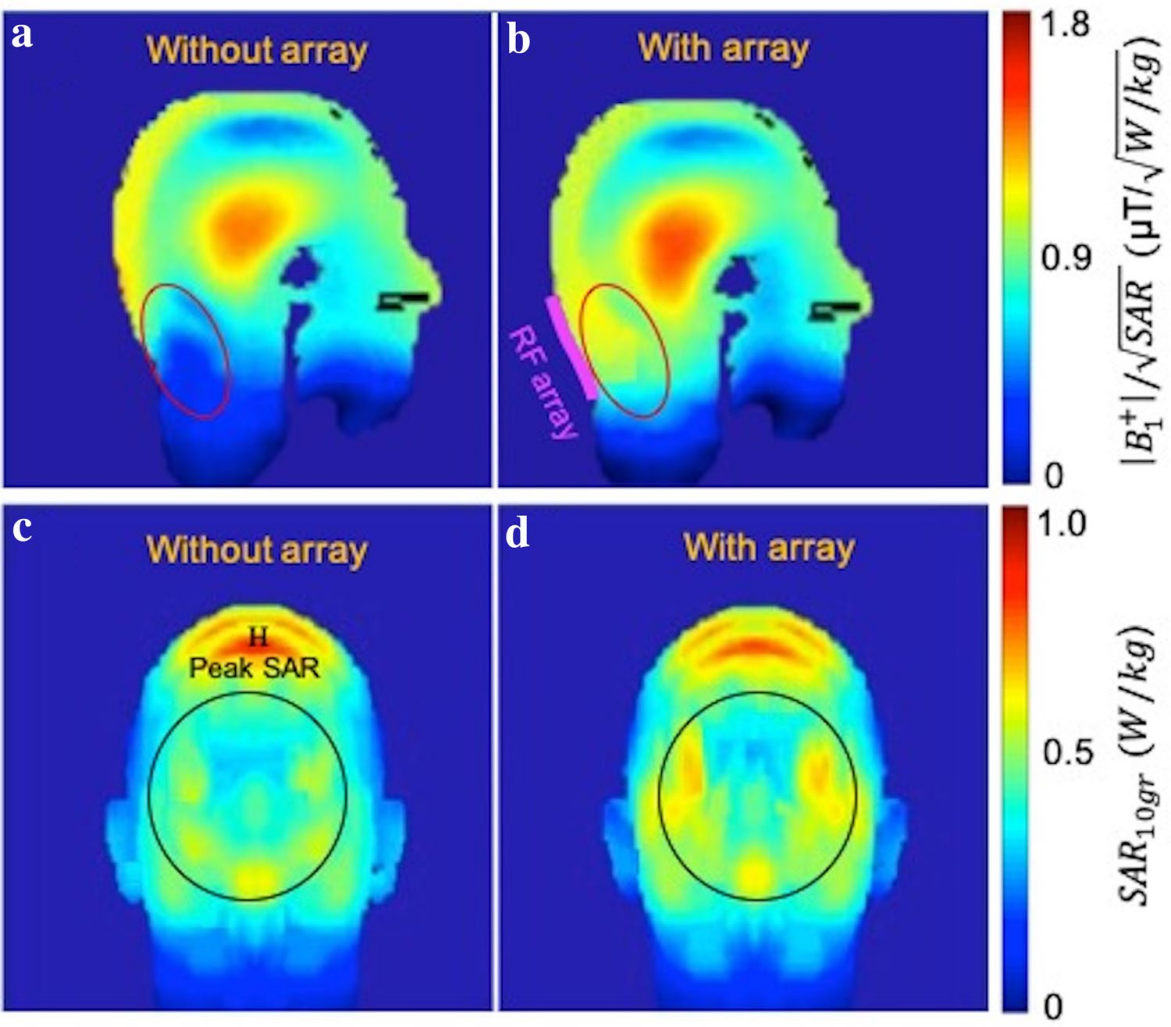
Simulated transmit field efficiency of a birdcage head coil (**a**) without the array and (**b**) in the presence of the array. Using the array resulted in improved transmit efficiency by factor of 3 in caudal regions of the brain when compared with the cases with no array. Simulated 10 gr local SAR distribution of the head coil without the array (**c**) and in the presence of the array (**d**). In the presence of the array the local SAR increased 24% at the area covered by the array indicated by a circle; and the peak SAR decreased %9 in the presence of the array.

The simulation results showed improved transmit efficiency ($\left|{B}_{1}^{+}\right|/\sqrt{SAR}$) at the regions covered by the array. The transmit efficiency was enhanced significantly in the ROI, as compared to the case without the array (Fig. 3a,b). The RF array resulted in 1.8-fold improvement in transmit efficiency in posterior brain and cerebellum regions (red ellipse).

Maximum 10 gr local SAR values were simulated using the human Duke model from the library of the CST (Computer Simulation Technology Microwave Studio). SAR distribution was computed in the birdcage coil without and in the presence of the array (Fig. 3c,d). The results showed that the SAR increased 24% at the location which the array was placed (encircled area), although the transmit efficiency was improved by 1.8-fold. Peak SAR (indicated with the H letter in Fig. 3c) decreased in the presence of the array by 9%.

### RF array prototype

A single BCSRR was fabricated using the preferred design parameters found in EM modeling. Parameter optimization was performed to obtain efficient electrical characteristics. The fabrication process included the following steps: (i) the first copper layer of SRR was patterned on one side of a flexible dielectric substrate (Kapton^®^ polyimide films, DuPont™), and (ii) a second copper layer of SRR was patterned on the other side of the substrate with 180° rotation but in the same axis as the first layer. The resulting geometrical parameters used for the BCSRR fabrication were: $D=50 \; \mathrm{mm}, d=200 \; \upmu \mathrm{m}, W=3 \mathrm{mm}, g=16 \mathrm{mm}.$

The built-in distributed capacitance between two layers in a BCSRR was used for fine frequency tuning. Changing the conductor length can affect the capacitance and inductance values, and consequently the operating frequency.

**Figure 4:**
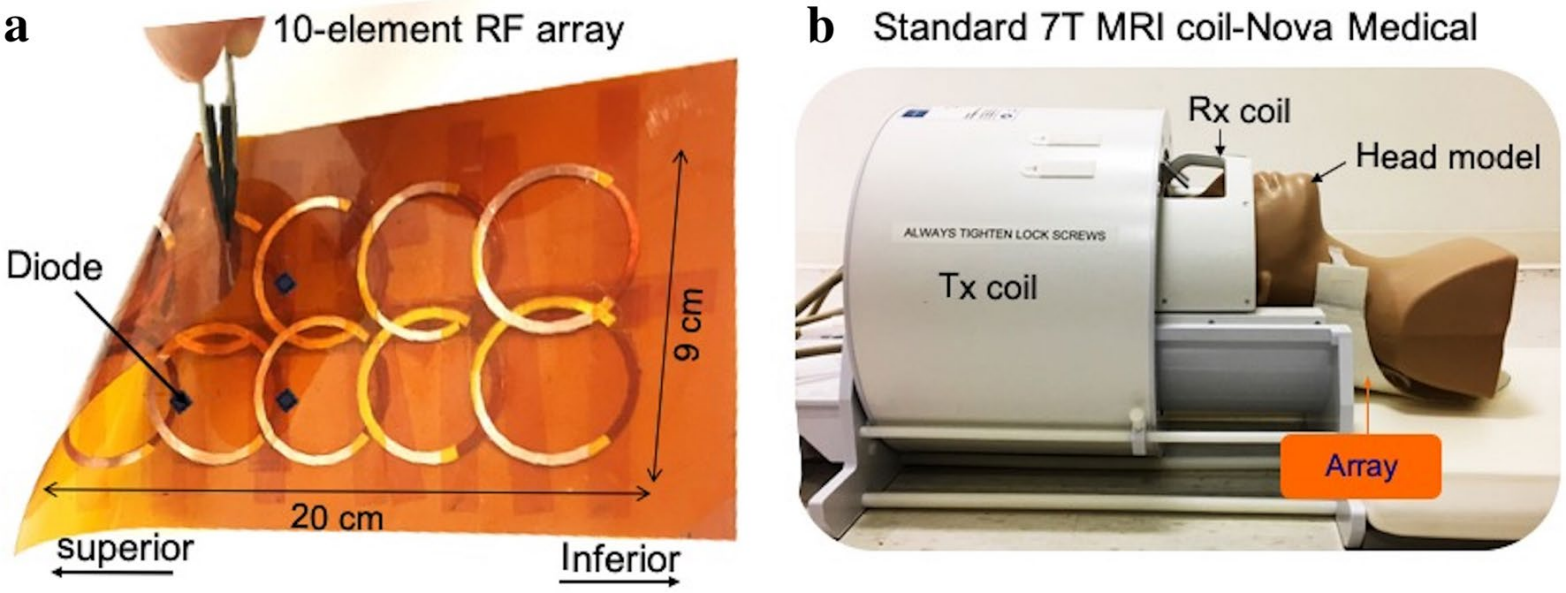
The prototyped RF array. (**a**) Prototype of the 10-element wireless passive RF resonator array constructed of 10 BCSRRs. Upon the substrate (Kapton), whose flexibility is shown by the instrument in the upper left, 10 SRRs were patterned on one side and 10 aligned but counter-oriented versions were applied to the other side. Critical overlapping distance (0.76D mm center-to-center) was used to reduce the inductive coupling between elements. Strongly coupled elements mainly in superior position were detuned using antiparallel cross diodes. (b) The picture of a standard head coil used in our MRI experiments and position of the array relative to the coil and a head model.

For the RF array fabrication, 10 SRRs (first layer) with 0.76*D* mm center-to-center overlapping distance were patterned on one side of a single piece of a dielectric substrate (Kapton), followed by patterning of another layer of 10 SRRs (second layer) on the other side, where first and second layers were aligned counter-oriented. The total dimension of the array is 9 cm × 20 cm (Fig. 4a).

The critical overlapping based on the loop center-to-center distance (0.76*D*) between neighbouring elements served to reduce the inductive coupling between elements. In order to avoid the RF over-flipping and boosting the absorption RF energy during the transmission, some of the resonators were decoupled from RF excitation using antiparallel cross diodes (Macom_,_ Newport Beach, CA, USA), specifically the resonators which were inside the MRI coil in strong coupling position with the RF excitation. The resonators with diodes enhanced only the receive signal. The circuit model of a decoupled resonator encircled with a blue dashed line is shown in Fig. 2b. The coupling level was evaluated using ${B}_{1}^{+}$ mapping method, which will be explained later.

The array extended out of the coil (Fig. 4b), extends the region of usable ${B}_{1}^{+}$ coverage outside of the volume coil.

### Bench-top measurements

Although the required design can be achieved through a simulation analysis, it can also be tuned on the bench-top based on transmission and reflection coefficients measurements. Specifically, to obtain the minimum decoupling condition, *y* should be adjusted such that the frequency with minimum transmission coefficient (${S}_{21}$) is equal to the tuned frequency (312 MHz).

**Figure 5:**
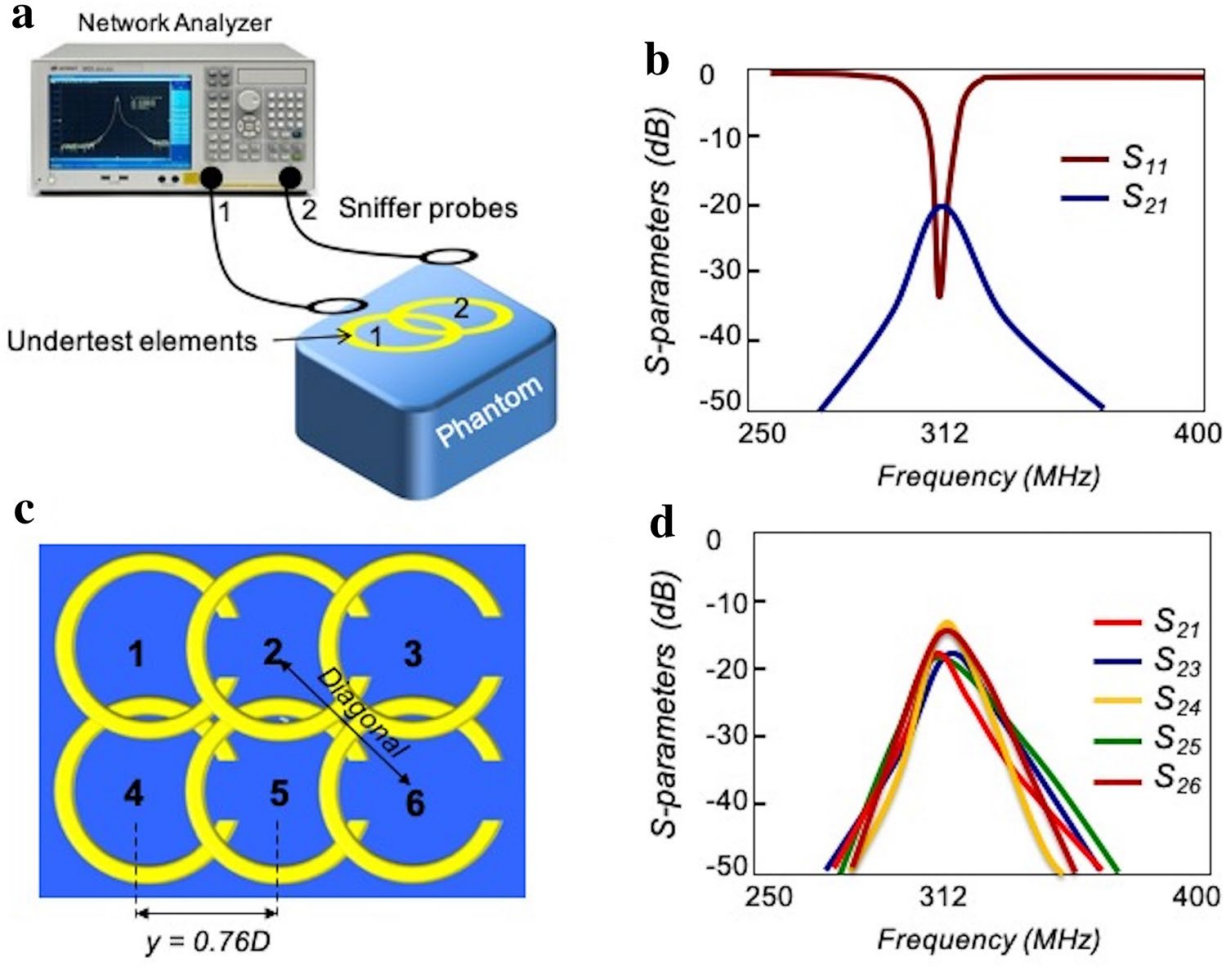
Bench-top test. (**a**) Bench test setup to measure the coupling between two resonators. (**b**) Measured S-parameters for two decoupled resonators. S21 shows about − 20 dB decoupling level, the symmetric behavior of S11 around *f*_*0*_ = 312 MHz also proves the fact. (**c**) All the inline elements were separated by critical overlapping technique. Diagonal elements are slightly coupled due to their geometrical positions. (**d**) Measured S-parameters show the coupling values of the resonator number 2 with three others inline neighbouring (1, 3, and 5) and diagonal (4 and 6) resonators. Measured S21, S23, and S25 show coupling values below − 16 dB, while S24 and S26 show coupling value about -14 dB.

An array of two decoupled resonators illustrating the bench-top experiments (Fig. 5a) used for S-parameter measurements plotted in Fig. 5b. As expected, the critically overlapped ($y=0.76 D$) resonators are strongly decoupled from each other, with a ${S}_{21}$ of − 20 dB. The strong decoupling also makes the reflection coefficient (${S}_{11}$) plot symmetrical around the tuned frequency.

A 10-element array consisting of 10 resonators (critically overlapping 2 $\times$ 5 matrix) was further built resulting in which two kinds of significant coupling exist between the elements: (i) coupling between inline neighbouring elements; and (ii) coupling between diagonal elements (Fig. 5c). Decoupling between elements was examined in the presence of the phantom by ${S}_{21}$ measurements between pairs of elements while all other neighbouring elements were detuned.

Figure 5d shows the coupling levels of a single element (#2) relative to the adjacent inline neighbouring (#1, 3, 5) and diagonal elements (#4, 6). In the array construction, geometrically overlaps were adjusted to achieve an acceptable decoupling level (< − 16 dB) between inline elements. Diagonal elements showed a decoupling level of about − 14 dB.

The loaded and unloaded Q-factor for the resonators was calculated and the average loaded Q-factor of 12 and average unloaded Q-factor of 18 were calculated. We also tested the effect of bending on the 2-element array by 45°. Bending the array in the middle did not significantly change the S-parameters.

### Heating experiment

Safety testing following a suitable modification of ASTM standards was performed to ensure tissue heating remained well below safety limits. After 15 min of RF transmission, a maximum temperature increases of 0.55 °C and 0.71 °C were experienced at the decoupled (detuned by diode) and coupled (tuned) RF resonators, respectively. While a temperature rise of 0.5 °C was recorded from the control probe. Corresponding SAR gains of 1.11 for the detuned array and 1.45 for the tuned array were calculated relative to the counterpart point in control (without array) set up. Therefore, for safe scanning, artificially reduced SAR limits of 88% and 70% must be adhered to for detuned and tuned arrays, respectively. In Supplementary C, Supplementary Tables 1 and 2 summarize the measured temperatures and calculated SARs at various points in the vicinity of the array. The temperatures at each position remained increased for several minutes after RF transmission was turned off. This indicates that thermal convection in the gel was low, suggesting that the gel experiment exaggerated heating above what would be seen in vivo where blood-flow enhances convective cooling. Additional safety analyses in the presence of the array were performed at various positions relative to the phantom and the head coil which are shown in Supplementary C.

### In vivo brain MRI

**Figure 6:**
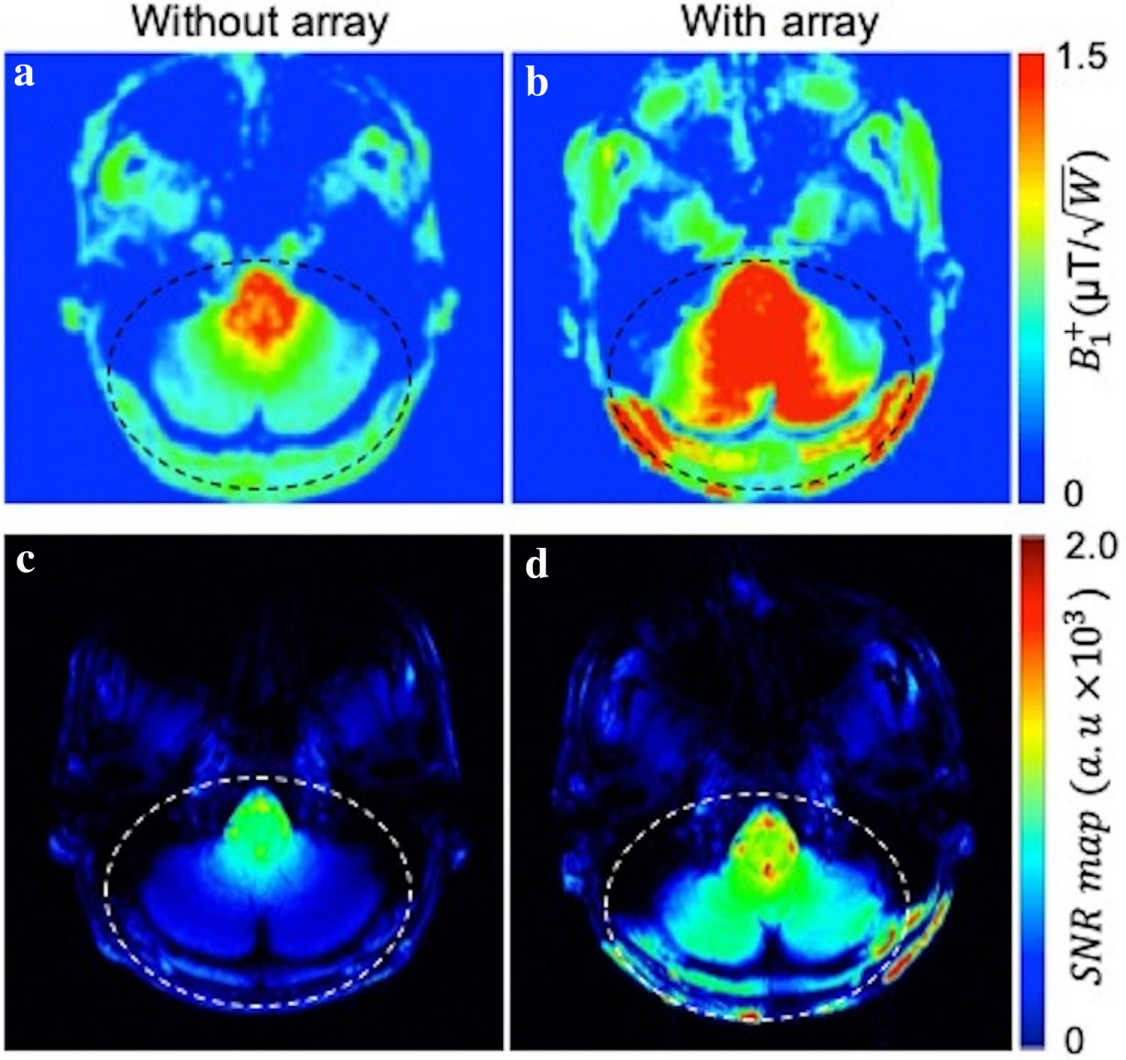
Experimental ${B}_{1}^{+}$ maps without (**a**) and with (**b**) the array show an improvement with the RF array in the inferior regions of the brain. Average ${B}_{1}^{+}$ enhancement of 1.8 $\pm$ 0.2 was calculated across all subjects in the cerebellum and brainstem. Experimental SNR maps without (**c**) and with (**d**) the array for one subject show that placing pads of the array at the back of the neck improves SNR in the cerebellum, neck muscles, and brainstem.

Figure 6a,b show measured in vivo axial transmit field (${B}_{1}^{+}$) maps obtained with and without the RF array in one of the five human subjects. ${B}_{1}^{+}$ maps are calculated using the turbo-FLASH based method with the same input power. The average improvement factor of about 1.8 $\pm$ 0.2 is measured over the ROI (dashed ellipse) in five human subjects.

Figure 6c,d show the experimental SNR maps without and in the presence of the RF array in the axial plane. The array improves the SNR by an average factor of 2.2 in the ROI including the cerebellum and brainstem. Supplementary Figure S2 showed ${B}_{1}^{+}$ maps and SNR maps acquired in a phantom.

In Supplementary Fig. S3, ex vivo MRI experiments at 7T conducted on 3 postmortem musk ox brains (in the context of an unrelated research project) using a Nova 1Tx/32Rx head coil in conjunction with the wireless RF array showed an average enhancement factor of 2.

**Figure 7:**
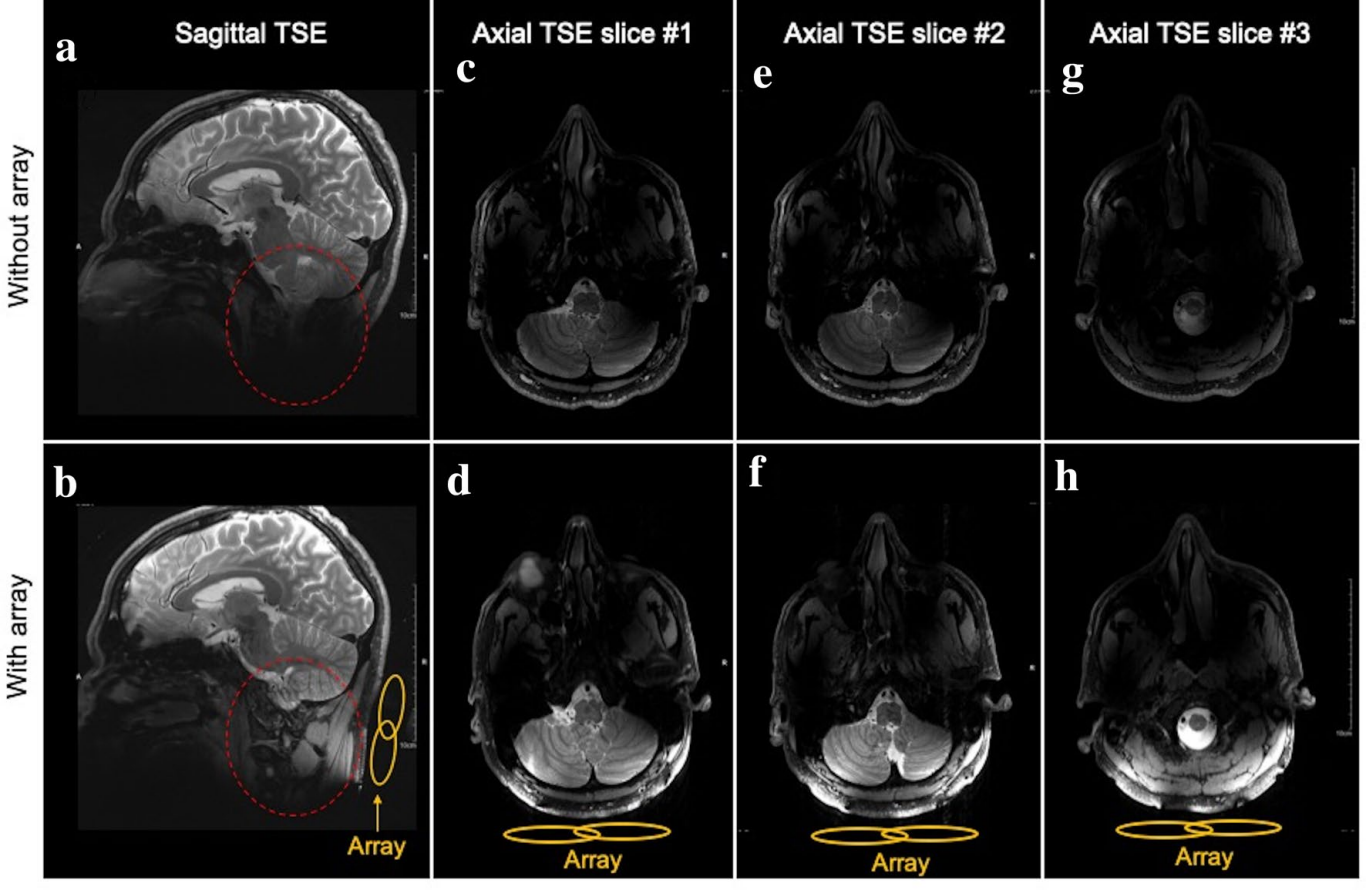
In vivo brain MRI at 7T. Sagittal T2-weighted TSE images obtained without (**a**) and with (**b**) the RF array show significant SNR and CNR improvement in the inferior regions in the presence of the array. In particular, the cerebellum, brainstem, and neck muscle are more clearly visible using the RF array. Axial T2-Weighted TSE slices obtained at various locations in the lower brain. Images obtained in slice #1 (**c**,**d**) show that placing the RF array results in significant improvement in ${B}_{1}^{+}$ uniformity and SNR in the cerebellum and brainstem. Slice #2 (**e**,**f**) and slice #3 (**g**,**h**) obtained at the physical border and 2 cm extended outside of the border, respectively. These images show that using the RF array extends the anatomical coverage of the head coil visualizing vertebral artery and spinal cord, which are hardly visible without the array.

In vivo MRI feasibility of the RF array was studied in five human subjects using TSE and GRE sequences. The array was placed behind the neck covering the posterior fossa, where the ${B}_{1}^{+}$ efficiency and signal sensitivity are intrinsically poor. Figure 7a,b show the sagittal T2-weighted TSE images improved visibility of the cerebellum, brainstem, and cervical vertebrae (red dashed circle) in the presence of the RF array. Also, in Supplementary Fig. S4, the axial T1-weighted GRE images obtained with and without the array focusing on posterior fossa show that the presence of the array improves the image uniformity and visibility of the cerebellum and the brainstem.

Figure 7 also shows the axial T2-weighted TSE images obtained at various slices locating at the lower brain without and with the RF array shown. Slice #1 (Fig. 7c,d) includes images focusing on the posterior fossa demonstrating significant ${B}_{1}^{+}$ efficiency and SNR enhancement in this region. Slice #2 (Fig. 7e,f) and Slice #3 (Fig. 7j,h) consist image obtained approximately at the physical border of the coil and 2 cm away from the border (extended out in the MRI z direction), respectively. The presence of the array extends the spatial coverage of the coil allowing visualizing spinal cord and vertebral artery, which are barely detectable using the head coil without the array.

Axial TSE 0.7 mm in-plane resolution images of the cerebellum and brainstem demonstrate exquisite anatomical detail with excellent gray matter/white matter contrast. An average CNR enhancement of 52% and 58% between gray matter and white matter in the cerebellum was calculated in TSE and GRE images, respectively.

## Discussion

We have proposed and validated an effective and simple design of a passive RF array to improve transmit efficiency and signal sensitivity in MRI systems. This array works in conjunction with the MRI coils by placing it over the ROI and requires only to be tuned to the desired operating frequency. A wireless passive RF resonator array providing solution for lack of spatial coverage and ${B}_{1}^{+}$ inhomogeneity problems in standard MRI coils was constructed and tested. The flexible and distributed architecture made the array advantageous in tunable and size-adjustable for various MRI applications. The array did not used any lumped element components, which allowed to maintain flexibility and may also prevent creating severe SAR hot spots. To prevent ${B}_{1}^{+}$ over-flipping, elements inside the MRI coil which were in strongly coupled position with RF excitation (Fig. 2e) were decoupled from RF excitation using anti parallel diode. Elements positioned in the inferior of the coil left tuned to enhance the signal in both transmit and receive phases. In the Supplementary B, we showed that to obtain optimized transmit efficiency in the presence of the resonator the off-resonance frequency of the resonators should be adjusted 5% above the Larmor frequency.

Several methods have been described to improve transmit field efficiency in MRI, such as pTx coils and passive RF shimming techniques^28, 30^. In this study we focused on using a wireless RF array in conjunction with a standard head coil to improve the whole-brain MRI at 7T by improving the receive signal sensitivity and transmit efficiency in the brain, particularly in the posterior fossa. The transmit and receive inductive coupling of the RF array with the RF excitation and magnetization vector not only improve the transmit efficiency and receive sensitivity but also extend the anatomical coverage to visualize regions inherently outside of the coil. Note that elements with diodes were decoupled from RF excitation, therefore they only enhanced the receive signal. Elements without diodes were inductively-coupled in both transmit and receive phases, therefore they improved transmit efficiency and signal sensitivity.

We used a commercial head coil to demonstrate the improvement in coil sensitivity. The coil suffered from limited sensitivity at the posterior fossa, which was improved by placing the RF array near inferior regions of the head. In addition to non-uniformity of the high RF field at UHF, the increased vulnerability of UHF imaging to susceptibility-induced image distortions near air-filled cavities is also challenging, especially in cerebellum imaging^22, 47^ where physical location in the posterior cranial fossa and anatomical diversity, combined with its small size, presents challenges for UHF MRI. Susceptibility artifact in particular was not addressed in this study.

The array performance was evaluated using EM simulations, bench tests, and MRI experiments. We demonstrated, in both simulations and experiments, that the SNR and the transmit efficiency of a commercial head coil at 7T can be improved in the skull base and cerebellum using a passive RF array. This enhancement in SNR was used for improving whole brain imaging, where the standard coil is limited to image due to poor transmit and receive sensitivity.

SAR distribution of the standard coil was manipulated in the presence of the RF array. The 10 gr local SAR increased 24% with array in the location which the array was presented, producing greater transmit efficiency at this location; but the peak local SAR decreased 9% when the array was used. Transmit inductive coupling between the RF excitation and some of elements in the array could be considered as a major reason of SAR amplification. A mitigation in the local peak SAR can be explained by a variation in the global RF energy distribution over the object in the presence of the array. Temperature tests under high SAR MRI sequences also reported a maximum local SAR gain of about 1.45 and 1.11 in the presence of the tuned and detuned elements, respectively.

Considering the previously mentioned enhancement in local SAR, the increase in transmit efficiency per square root of maximum SAR, experimental in vivo ${B}_{1}^{+}$ maps used in evaluating RF coil transmit efficiency, was a factor of 1.8 $\pm$ 0.2. In practical terms the increase in transmit efficiency means that the amplitude or duration of the transmitted RF pulse can be reduced by the equivalent factor. The average in vivo SNR enhancement of 2.2-fold was calculated using the RF array, which corresponds to a decrease in total experimental scan time for a constant SNR. Note that the reference voltage was kept constant for both with and without array cases, we did not re-calibrate the reference voltage between array and no-array cases to disentangle the effect of receive amplification from increases in flip angle (FA) in the SNR. The experimental SNR analysis scaled by the ${B}_{1}^{+}$ determined that 2.2-fold SNR enhancement was mainly due to the improved transmit efficiency (due to FA amplification) and partially (33%) due to received-only coupled sensitivity improvement. In vivo images showed that sensitivity, contrast, and image uniformity were enhanced in the presence of the RF array, which resulted in improved visibility in the inferior region of the human brain extended outside of the coil (Fig. 7).

This study has demonstrated the promise of a novel flexible and compact RF resonator array structure, which can improve MRI performance in regions where transmit efficiency is reduced due to wavelength effect. This new flexible RF structure is the first broadside-coupled SRR array, which can be integrated into MRI coils. The utility of the RF array acquiring in vivo human brain images on a commercial 7T MRI system was also shown. In this demonstration we targeted the posterior and caudal brain including cerebellum and brainstem, regions with limited coverage due to coil structure. Experimental results showed that the RF array can enhance the local transmit field and improve the receive signal sensitivity at the localized region. The proposed RF resonator array, constructed of a very simple substrate, can be considered a low-cost and time-stable alternative to other passive RF-shimming techniques. Although this work demonstrated 10-element RF array in brain MRI at 7T, where the ${B}_{1}^{+}$ inhomogeneity is more severe, it can be modified to be implemented in other MRI applications when the transmit efficiency and receive sensitivity improvement are required.

## Methods

### RF array modeling

When designing a resonator, it is desirable to control the capacitance and inductance to tune the resonator^48, 49^. The built-in distributed capacitance between the metal layers in BCSRR structure used to tune the resonator avoids the need for any lumped element capacitance. In addition, distributed capacitance spreads out the electric field along the resonator and prevent the resonator from creating severe SAR hot spots^50^. A series of electromagnetic (EM) simulations [Computer Simulation Technology Microwave Studio (CST), Germany] was performed to investigate the effects of design parameters on the electrical properties of a single BCSRR. Optimized resonator geometry was used for the array modeling; further details are provided in Supplementary A.

A coupling an array of resonators to a volume coil is different from the coupling a single resonator to a volume coil. Since all the resonators in the array are inductively coupled through the volume coil, therefore, the array interaction with the coil must be considered as well in global ${B}_{1}^{+}$ efficiency and signal sensitivity. We conducted EM numerical simulations (CST) to evaluate the EM field distribution of a head coil in the presence of the RF array. We modeled a head-sized birdcage coil (22 cm in diameter and 25 cm in length), similar to the transmit head coil (Nova Medical, Wilmington, MA, USA) used in the experiments. The coil had 16 rungs connected at each end to two end rings and shielded by an open cylinder (23 cm in diameter and 27 cm in length). The coil was tuned by lumped capacitors distributed at the end-ring gaps and matched to 50 Ω using a single capacitor at each port, placed in series with an ideal voltage source with a 50 Ω internal resistance. The CST time domain solver was used to evaluate the ${B}_{1}^{+}$ field distribution in the birdcage coil containing the virtual voxel head model (Duke) at 7T.

To evaluate the RF array effect on the SAR distribution, we calculated 10 gram (10 gr) average SAR for with/without the array using the same birdcage coil containing “Duke” head model. Time-averaged SAR values were calculated by finding the time derivative of the incremental energy, absorbed by an incremental 10 gr mass of tissue. All resulting simulated SAR values were compared with the corresponding limits (10 W/kg for maximum local SAR and 3.2 W/kg for head average SAR) recommended by the FDA and IEC^51^. The SAR distribution was used to estimate the possible hot spots for the heating test.

### Electrical bench test

All elements were tuned to ${f}_{0}^{ }=312 \; \mathrm{ MHz},$ 5% above the Larmor frequency at 7T, 297 MHz to obtain optimized transmit efficiency in the presence of the resonator (Supplementary B) while loaded with the cylindrical saline phantom (15 cm in diameter and 30 cm in height; relative permittivity: 75; conductivity: 0.60 S/m). Tuning was assessed by measuring the reflection coefficient (${S}_{11}$) using a single sniffer probe in a calibrated vector network analyzer (VNA, E5071C, Agilent Technologies, Santa Clara, CA, USA). Detuning performance of the resonators (with antiparallel diode) was measured as the change in the scattering parameter ${S}_{21}$ of a loosely coupled double pick-up probe. The double pick-up probe consists of two overlapped sniffer loops made out of semi-rigid coaxial cable with a gap in the shield placed symmetrically in the middle of each loop^52^. The sniffer loops were overlapped to the extent required for inductive decoupling. Sniffer loop #1 transmits RF energy to the resonant element under test and sniffer loop #2 functions as a pick-up coil detecting currents excited in the resonant element under test. Q-factor of each element was calculated as ${f}_{0 }/\Delta f$, where $\Delta f$ is the FWHM bandwidth of the measured ${S}_{21}$ using the double pick-up probe. Decoupling between neighboring elements was adjusted to null their mutual inductance by moving those tuned elements towards each other while measuring the ${S}_{21}$ parameter between the elements. ${S}_{21}$ interaction between the pairs of elements was monitored using two independent sniffer loops: one sniffer loop connected to port 1, transmitting RF power, and coupled to the first element under test; and the other sniffer loop connected to port 2, receiving the induced RF power, and coupled to the second element under test (Fig. 5a). When measuring the decoupling between an adjacent pair, all other unused elements of the array were detuned.

### Experimental heating test

To evaluate the presence of the RF resonator array in causing a possible safety concern in a mode of heating, temperature measurements were conducted in an MRI scanner following a suitable modification of ASTM standards^53^. The RF array was immersed inside an ASTM gel phantom (${\delta }_{gel}$ = 0.50 S/m, ${\varepsilon }_{gel}$ = 77, heat capacity = 4154 J/kg °C), in the location within the volume coil where the array was expected to be during the human MRI experiments. The array was coated with a thin layer of plastic to avoid the direct contact of the array with the gel. The assembly was placed inside the head coil and was scanned for 15 min with a high SAR turbo-spin-echo (TSE) sequence (repetition-time (TR) = 500 ms, echo-time (TE) = 11 ms, Flip angle (FA) = 120°, bandwidth = 277 Hz/pixel, FOV = 16 cm × 23 cm, matrix = 256 × 256, average = 32, slice thickness = 10 cm). RF excitation was performed using a Nova 1Tx/32Rx head coil (Nova Medical, Wilmington, MA, USA) in a 7 T MRI scanner (Magnetom, Siemens Healthcare, Erlangen, Germany). Temperature was measured using four fiber-optic temperature probes (LumaSense Technologies, Santa Clara, CA) located at the possible hot spots estimated using SAR simulations in the presence of the array. Baseline temperatures were recorded before RF transmission, and temperature changes were measured during scanning. SAR was calculated as; $SAR={C}_{hc}(dT/dt)$, where ${C}_{hc}$ is the heat capacity, *T* is the temperature and *t* is the time.

We used the exact same location of the probe when studying the temperature changes occurring with and without the array. We visually examined the location of the probes relative to the RF array, immediately before and after the heating assessment because slight variability in probe positions relative to the array can lead to significant variations in the measured temperature.

### In vivo MR imaging

In vivo ${B}_{1}^{+}$ and SNR maps were calculated in the human brain with and without the resonator array. The array was placed behind the neck covering the posterior fossa. The flexible and thin structure of the array allowed it to be placed on the curved surface of the back to fully cover the ROI. ${B}_{1}^{+}$ maps were measured using the presaturation-prepared turbo-FLASH based method (MGH QA package) with acquisition parameters TR/TE = 2.7/1.2 ms, FA = 10, FOV = 16 cm × 21 cm, matrix = 256 × 256. The applied input power was the same (200 V) for with/without the array ${B}_{1}^{+}$ mappings.

The signal reception performance of the array was evaluated using SNR map calculations with/without the array. SNR maps were generated using the images obtained from two gradient-recalled echo sequences (GRE, TR/TR = 400/9 ms, FA = 5°, bandwidth = 977 Hz/pixel, FOV = 16 cm × 21 cm, matrix = 256 × 256), one with and the other without RF transmission (MGH coil QA package was used).

For in vivo imaging, five healthy adult volunteers (3 males, 2 females, age 30–45 years) were imaged on the 7 T whole body MRI scanner. MR images were acquired with and without the resonator array using a Nova 1Tx/32Rx head coil. The imaging sequence was a GRE sequence with parameters: TR = 100 ms, TE = 4 ms, FA = 10°, bandwidth = 977 Hz/pixel, FOV = 9 cm × 14 cm, matrix = 256 × 256. TSE images were also acquired with the parameters TR/TE = 3000/76 ms, FOV = 22 cm × 18 cm, flip angle = 120°, slice thickness = 2 mm, bandwidth = 977 Hz/pixel, matrix = 256 × 256, TSE factor = 15. The human experimental procedures were approved by the internal review board at the Icahn School of Medicine at Mount Sinai.

Contrast enhancement analysis was also conducted using calculation of contrast-to-noise ratio (CNR), formulated as: $CNR=\left|({S}_{ROI}-{S}_{REF})/{\sigma }_{N}\right|$, where ${{S}_{ROI}, S}_{REF}$ are the mean signal intensity of the ROI and signal intensities of the reference area, respectively. ${\sigma }_{N}$ is the standard deviation of the background noise.

All methods were carried out in accordance with the Icahn School of Medicine at Mount Sinai guidelines and regulations. All experimental protocols were approved by the Icahn School of Medicine at Mount Sinai Medical Ethics Committee. In vivo images of the brain were acquired from healthy volunteers after informed consent was obtained, in accordance with the guidelines of the Icahn School of Medicine at Mount Sinai Medical Ethics Committee.

## Supplementary Information

Supplementary Information.

## Data Availability

The data that support the findings of this study are available on request from the corresponding authors A.A. The data are not publicly available due to restrictions imposed by the local ethical committee as they contain information that could compromise the privacy of the research participants.
