# Supplementary material for: Improvement of magnetic resonance imaging using a wireless radiofrequency resonator array
Source: Sci Rep. 2021 Nov 29;11:23034. doi: 10.1038/s41598-021-02533-3 (PMC8630230; doi:10.1038/s41598-021-02533-3)
Supplement: Supplementary file 1 — Supplementary Information. [file 41598_2021_2533_MOESM1_ESM.docx]

^[[1]](#footnote-1)^

Improvement of Magnetic Resonance Imaging Using a Wireless Radiofrequency Resonator Array

Akbar Alipour^a^**,* Alan C Seifert^a^, Bradley N Delman^b^, Philip M Robson^a^, Raj Shrivastava^c^, Patrick R Hof^d^, Gregor Adriany^e^, Zahi A Fayad^a^, and Priti Balchandani^a^

Supplementary Materials

1. ***Supplementary Figures:***

Figure S1


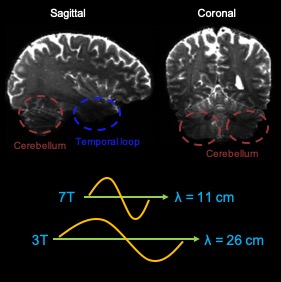


**Fig. S1**. RF wavelength (λ) effect and associated inhomogeneity problem at 7T, where λ (≅ 11 cm) is comparable with head dimensions. Excitation field inhomogeneity results in dark voids in the brain MRI at 7T, specifically in the lower brain and cerebellum as it is shown in sagittal and coronal images.

Figure S2


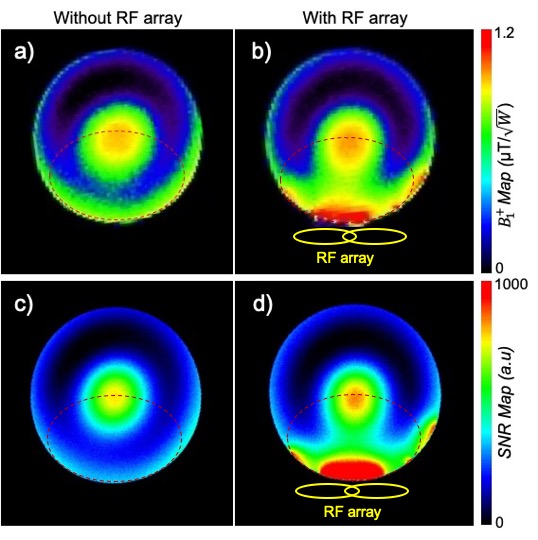


**Fig. S2**. $B_{1}^{+}$ maps were acquired in a phantom with and without the array on a 7T Siemens Magnetom whole-body scanner using a Nova 1Tx/32Rx head coil. The array was placed in the posterior position inside the coil and then the phantom was place inside the coil. $B_{1}^{+}$ maps were measured using the presaturation-prepared turbo-FLASH based method (MGH QA package) with acquisition parameters TR/TE=2.7/1.2 ms, FA=10, FOV= 16 cm × 21 cm, matrix= 256 × 256. The signal reception performance of the array was evaluated in the phantom using SNR map calculations with/without the array. All images were obtained on a 7T MR scanner using Nova 1Tx/32Rx head coil. SNR maps were generated using the images obtained from two gradient-recalled echo sequences (GRE, TR/TR= 400/9 ms, FA= 5°, bandwidth= 977 Hz/pixel, FOV= 16 cm × 21 cm, matrix= 256 × 256), one with and the other without RF transmission (MGH coil QA package was used). Phantom $B_{1}^{+}$ and SNR mappings. (a, b) Measured transversal transmit field ($B_{1}^{+}$) maps in the phantom with/without the RF array show that placing the array inside the coil results in an average 2-fold improvement in the transmit efficiency in the ROI encircled with dashed red. $B_{1}^{+}$ maps were normalized by the input power. (c, d) Experimental SNR maps in the phantom with/without the RF array show that the array provides an average 2.4-fold enhancement in the ROI encircled with dashed red in **a**.

Figure S3


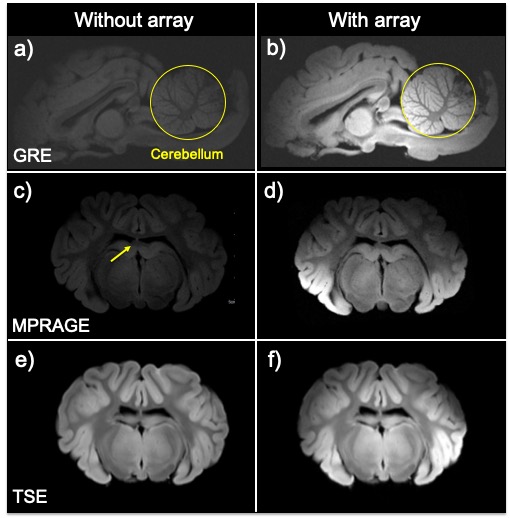


**Fig. S3**. *Ex vivo* MR imaging was performed in three formaldehyde-fixed adult musk ox brains obtained postmortem from animals that died of natural causes in the context of an unrelated research project. The brains were secured inside cylindrical containers (12 cm in diameter and 17 cm in length) filled with fixative solution (400 ml of 10% neutral buffered formalin). The assembly was then subjected to vacuum for 30 min to remove the air bubbles. The brains were imaged on 7T Siemens Magnetom whole-body scanner using a Nova 1Tx/32Rx head coil. The RF array was placed at the posterior position of the head coil and specimen placed on top of the array. Images were obtained with/without the resonator array. First column (left) images (a, c, and e) were obtained without the array and second column (right) images (b, d, and f) were obtained with the array on a 7T MRI scanner. Sagittal small tip angle GRE images without array (a) and with array (d) shows significant SNR and CNR enhancement in the whole brain when using the array. In particular, inferior central brain and cerebellum are more clearly visible in the presence of the array with about 2.6-fold SNR improvement. Comparison of T1-weighted coronal MPRAGE images (second row, c and d) shows signal and contrast improvement in the outer (cortical) regions of the brain and thalamus (indicated with a yellow arrow) when using the array. TSE images obtained without (e) and with (f) the array show signal enhancement of about 2-fold in the thalamus.

Figure S4


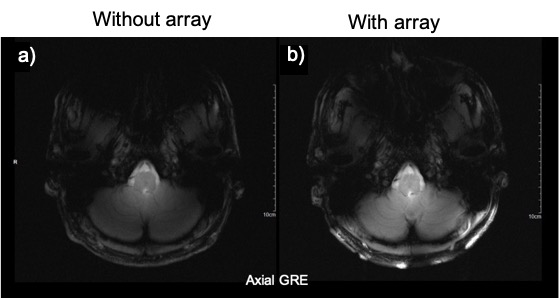


**Fig. S4**. *In vivo* brain MRI at 7T. Axial GRE images obtained without (c) and with (d) the RF array show significant SNR and CNR improvement in the inferior regions in the presence of the array. In particular, the cerebellum, brainstem, and neck muscle are more clearly visible using the RF array.

1. ***Supplementary A***

Electrical characteristics (resonance frequency ($f_{0}$) and Q-factor) of a resonator are given by Eq. (1) and Eq. (2), respectively:

$w_{0}=\frac{1}{\sqrt{LC}}$ (1)

$Q= \frac{2\pi f_{0}L}{R}$ (2)

where$L$ is the effective inductance, $R$ is an AC resistance of the structure, and $C$ is the effective capacitance of the overall structure. The approximate effective inductance, capacitance, and resistance of the given design can be formulated as ^46^:

$L=2.54\mu D\left\{ \ln\left( \frac{2.07}{\rho} \right)+0.18\rho+0.13\rho^{2} \right\}$ (3)

$C=\varepsilon_{0}\varepsilon_{r}\frac{A}{d}$ (4)

$R= \frac{2l}{T\sigma\delta(1-e^{{-b}/\delta})}$ (5)

where $\mu$ is the permeability of the copper, *D* is the average diameter $\left( D={(\left( D_{o}+D_{i} \right)}/2 \right)$, $\rho$ is the fill ratio $\left( \rho=\left( D_{o}-D_{i} \right)/\left( D_{o}+D_{i} \right) \right)$, $D_{o}$ is the outer diameter, $D_{i}$ is the inner diameter, $\varepsilon_{0}$ is the permittivity of the free space, $\varepsilon_{r}$is the relative permittivity of the dielectric substrate, *A* is the parallel plate surface area, *d* is the distance between the consecutive layers (dielectric thickness), *W* is the metallization (copper) width, $l$ is the path length of the metal trace, $b$ is the copper thickness $\left( 35 \mu m \right)$, $\sigma$ is the conductivity of the copper, and $\delta$ is the skin-depth of the copper. The skin-depth, $\delta=\sqrt{2/{2\pi f_{0}\mu\sigma}}$ can affect the AC resistance ($R)$ consequently the Q; smaller skin-depth (due to higher frequency) causes increasing AC resistance of the conductor. To maximize the Q, the metal thickness should be at least two times higher than the skin-depth ^45^. In this study, $\delta=3.8 \mu m$ (at 297 MHz) and metal thickness = $35\mu m$.

The resulting geometrical parameters used for the BCSRR fabrication were: $D_{o}=50 mm, D_{i}=44 mm, D=47 mm, \rho=0.064, d=200 \mu m, W=3 mm, g=18^{\circ}, l=152 mm, A=444 mm^{2}.$

## Single BCSRR Modeling

The inductive coupling between the transmit magnetic field and the RF resonator generates an additional magnetic field that manipulates the total magnetic field. In addition, in the receive phase, coupling between the magnetization vector (M) and the resonator enhances MR signal. The resonator inductance $(L)$ and capacitance $(C)$ play a main role in controlling the Q-factor and tuning the frequency. $L$ also has an important role in inductive coupling levels.

The inductance and capacitance values of a single resonator are controlled by the design parameters *D,* $W$*,* $g$*,* and *d*. The effect of design parameters was numerically analyzed for different values. Results for five diameter (*D*) values, with *d*, $g$, and *W* kept constant ($d=200 \mu m, g=18^{\circ}, W=3 mm$), showed that $L$and $C$ were increased as *D* increased (Fig. SA.1a). Increasing diameter is associated with extending the capacitive area and conductor length, which results in increased $L$ and $C$ values, respectively.

The gap width ($g$) effect was studied with other parameters kept constant ($d=200 \mu m, D/W= 50/3 mm$). As $g$ increased both $L$ and $C$ decreased, since the capacitive area and conductor length decreased (Fig. SA.1b).

The dielectric thickness (*d*) effect was evaluated, with *D*, $g$, and *W* kept constant ($D/W=50/3 mm, g=18^{\circ}$). The effective capacitance $C$ decreased by increasing *d*, as the capacitance value is inversely proportional with the dielectric thickness (Fig. SA.1c). The effective inductance $L$ did not show a significant change by *d* variations.

Results for conductor width (*W*) as the other parameters keep constant ($d=200 \mu m, D= 50 mm, g=18^{\circ}$), showed that $C$ increased and $L$ decreased as *W* increased (Fig. SA.1d). Increasing *W* results in higher capacitive area, consequently higher $C$ values. On the other hand, increasing *W* leads larger conductor cross-section for electrical charge flow, which resulted in lower $L$ values.


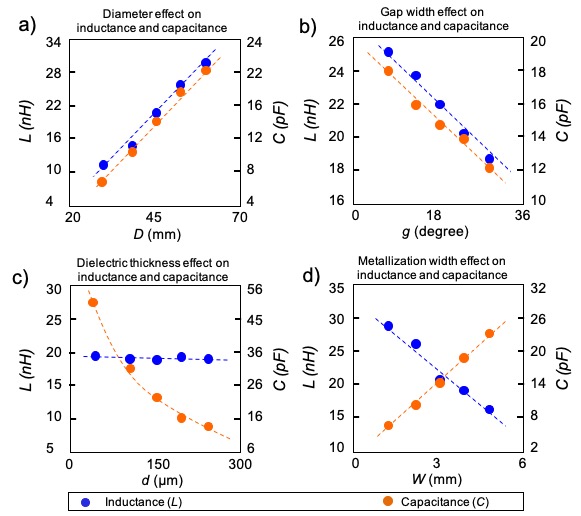


**Fig. SA.1**. EM simulation results detailing effects of the parameters *D*, *g*, *d*, and *W* on the resonator inductance (L) and capacitance ($C$). (a) Effect of diameter (*D*=27, 37, 47, 57, 67) with other parameters (*W* =3 mm, *d* = μm 200, and *g* = 18°) kept constant. As *D* increased, the $L$ and $C$ increased due to increasing the capacitive area and conductor length. (b) Effect of gap width (*g* = 6°, 12°, 18°, 24°, and 30°) with *D*, *W*, and *d* kept constant (*D*/*W*=47/3 mm, d=200 μm). The $L$ and $C$ decreased as *g* increased*.* (c) Effect of dielectric thickness (*d* = 50, 100, 150, 200, and 250 μm) with *D*/*W*=47/3 mm, *g* = 18°. $C$ decreased as it is proportional with the inverse of dielectric thickness. $L$ was not changed with *d* variations. (d) Effect of metallization width (*W* = 1, 2, 3, 4, and 5 mm). The $L$ decreased because of larger conductor cross-section and $C$increased due to increasing capacitive area as the metallization width increases, with other parameters kept constant (*D* = 50 mm, *g* = 18°, *d* = 200 μm).

In Figure SA.2, similar analyses evaluated the effect of the design parameters on $f_{0}$ and Q-factor. Increasing the $L$ and $C$ by increasing *D* resulted in decreasing $f_{0}$ (Fig. SA.2a). Q decreased as *D* increased, which can be explained by the dominant effect of frequency and skin effect decreasing. $f_{0}$ exponentially increased by increasing *g* (Fig. SA.2b), as $L$ and $C$ were decreased. Q was not affected by *g* variations. $f_{0}$ and Q linearly increased as *d* increased (Fig. SA.2c), this can be explained by decreasing $C$. $f_{0}$ exponentially decreased as *W* increased (Fig. SA.2d), because of the dominant effect of increased $C$. Q also decreased by increasing *W*. During each parameter evaluation, other parameters keep constant.

Ultimately, the following design parameters were used in 10-element array construction for efficient performance: $D=50 mm, d=200 \mu m, W=3 mm, g=18^{\circ}.$ These parameters were selected to: (i) optimize Q for sufficient signal enhancement and avoid RF over-flipping, (ii) keep the resonator size large enough to avoid wavelength effect.


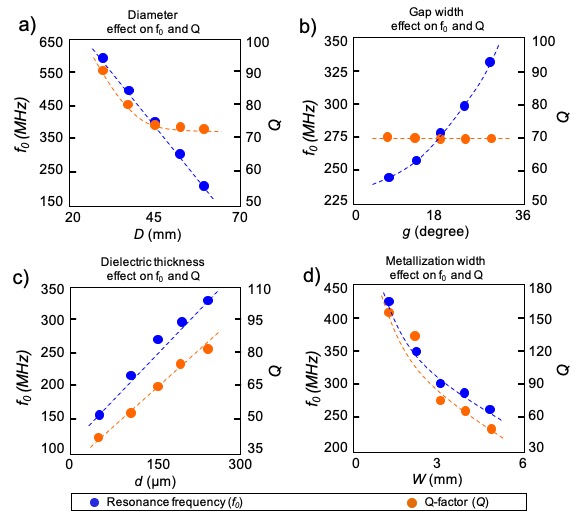


**Fig. SA.2**. EM simulation results detailing effects of the parameters *D*, *g*, *d*, and *W* on the resonator resonance frequency (*f_0_*) and Q-factor. (a) *f_0_* decreased as *D* increased, which can be explained by the dominant effect of increased L. *Q* showed a non-linear decreasing behavior as *D* increased. (b) *f_0_* exponentially increased and *Q* did not change as *g* increased. (c) *f_0_* and Q linearly increased as *d* increased. (d) *f_0_* and *Q* showed an exponential decreasing behavior as *W* increased. During each parameter evaluations other parameters were kept constant.

1. ***Supplementary B***

## Theoretical Background

In this study we used a commercially available Nova Medical 1Tx/32Rx birdcage head coil for RF transmission and MRI signal reception at 7T. An array consists of 10 broadside-coupled split-ring-resonators (BCSRR) was place inside the birdcage coil covering the base of the skull that it extended 6 cm out from the coil. Assume a single BCSRR, the circularly-polarized transmit magnetic field, $B_{rf}$ of the transmit coil inductively couples to the resonator, the reaction of the resonator with excitation filed leads to circulating current in the resonator, which results in a secondary magnetic field, $B_{re}$ (Fig. 1c) in the resonator vicinity.

Consider a circularly polarized magnetic field generated by the commercially available quadrature birdcage coil:

$\boldsymbol{B}_{rf}\left( t \right)= B_{1}^{+}\left( coswt \boldsymbol{i}-sinwt \boldsymbol{j} \right)$ (6)

$B_{1}^{+}$ is am amplitude modulation function and $w=2\pi f$ is the carrier frequency of the transmission.

The inductive coupling between the resonator and $\boldsymbol{B}_{rf}\left( t \right)$ results in a linearly polarized magnetic field ${(B}_{re})$ generated by a resonator, which can be expressed as:

$\boldsymbol{B}_{re}\left( t \right)=2B_{re}^{+} coswt \boldsymbol{i}$ (7)

Assume the angle between CP magnetic field lines and normal vector of the resonator is zero, therefore, from Faraday’s law of induction, the electromotive force $($ε$)$ generated by $\boldsymbol{B}_{rf}\left( t \right)$ is given:

$\epsilon=-\frac{d\phi}{dt}=(\pi r^{2})wB_{1}^{+}$ (8)

Where *r* is the radius of the resonator.

If the resonator is assumed as a series RLC circuit, the input impedance can be written as:

$Z_{re}=R\left[ 1+i\frac{wL}{R}\left( \frac{f^{2}-f_{0}^{2}}{f^{2}} \right) \right]$ (9)

where R represents the ohmic losses, L is the resonator inductance, $f_{0}$ is the resonance frequency of the resonator, $Q={wL}/R$ is the resonator Q-factor, and $f=1/{2\pi\sqrt{LC}}$ is the actual detuning frequency of the resonator. Assume $f=(f-f_{0})$ and consider $f$ is relatively small compared to $f_{0}$. The impedance can be simplified to:

$Z_{re}=R\left[ 1+i2\frac{wL}{R}\left( \frac{f}{f_{0}} \right) \right]$ (10)

The associated ohmic loss, R, is typically small, therefore the induced current on the resonator can be written as:

$I_{re}=\frac{\epsilon}{Z_{re}}\simeq\frac{\left( \pi r^{2} \right)B_{1}^{+}}{L}\left( \frac{f_{0}}{f} \right)$ (11)

The modulation magnetic field generated by the induced current at distance $z$ away from the resonator center is given by:

$\boldsymbol{B}_{re}\left( t \right)\simeq\frac{\mu(\pi r)B_{1}^{+}}{L\left( 1+\left( \frac{z}{r} \right)^{2} \right)^{\frac{3}{2}}}\left( \frac{f_{0}}{f} \right)coswt\boldsymbol{i}$ (12)

This linearly polarized field decomposes into two circularly-polarized fields. One a circularly forward-polarized field and the other a circularly reverse-polarized field, which mathematically can be written as:

$\boldsymbol{B}_{re}\left( t \right)\simeq\frac{\mu(\pi r)B_{1}^{+}}{2L\left( 1+\left( \frac{z}{r} \right)^{2} \right)^{\frac{3}{2}}}\left( \frac{f_{0}}{f} \right)\left[ coswt\boldsymbol{i-}sinwt\boldsymbol{j} \right]+\frac{\mu(\pi r)B_{1}^{+}}{2L\left( 1+\left( \frac{z}{r} \right)^{2} \right)^{\frac{3}{2}}}\left( \frac{f_{0}}{f} \right)\left[ coswt\boldsymbol{i+}sinwt\boldsymbol{j} \right]$ (13)

The first term represents the circularly forward-polarized field and the second one represents the circularly reverse-polarized field. We will neglect the second term, which has a negligible effect on the spin excitation, and consider only the forward-polarized field, which is more resonant with the spins and rotates in the same direction as the recessing spins.

Therefore, the total magnetic field at the distance $z$ from the resonator center is

$\boldsymbol{B}_{\boldsymbol{t}}^{\boldsymbol{+}}\left( t \right)\simeq\boldsymbol{B}_{rf}\left( t \right)+\frac{\mu\pi B_{1}^{+}}{2L\left( 1+\left( \frac{z}{r} \right)^{2} \right)^{\frac{3}{2}}}\left( \frac{f_{0}}{f} \right)\left[ coswt\boldsymbol{i-}sinwt\boldsymbol{j} \right]=\left[ 1+\frac{\mu\pi}{2L\left( 1+\left( \frac{z}{r} \right)^{2} \right)^{\frac{3}{2}}}\left( \frac{f_{0}}{f} \right) \right] B_{1}^{+}\left[ coswt\boldsymbol{i-}sinwt\boldsymbol{j} \right]$ (14)

$\mathrm{where}B_{1}^{+}$ is the original magnitude of $\boldsymbol{B}_{\boldsymbol{t}}^{\boldsymbol{+}}\left( t \right)$, when there is no resonator in place. Considering a resonator in this study tuned below the Larmor frequency ($f>f_{0})$, then the total magnetic field, $\boldsymbol{B}_{\boldsymbol{t}}^{\boldsymbol{+}}$ can be cancelled in the region effected by the resonator. Therefore, the desired off-resonance frequency,$f$ should be above the Larmor frequency to enhance the transmit field. In general, transmit field efficiency is lower at the inferior region of the coil and higher compensation may be required. We adjust off-resonance frequency 5% above the Larmor frequency to obtain optimized transmit efficiency in the presence of the resonator. The coupling between the resonator and the birdcage coil depends on the resonator orientation relative to the coil. Therefore, the transmit field profile of the resonator,$B_{re}$ depends on its relative orientation to the coil.

Inductive coupling of an array of resonators with the birdcage coil is more complicated than a coupling of a single resonator. All of the array elements are inductively coupled through the birdcage coil, therefore their interaction is considered well in global homogenization. To this end, we performed full-wave electromagnetic simulations for more complementary results.

1. ***Supplementary C***

*MRI Heating Test*

This test method covers measurement of RF induced heating on or near the passive wireless RF array and its surroundings during MRI. This test method is one required to determine if the presence of a passive wireless RF array may cause heating problem to the patient with the array during an MR procedure.

The amount of RF-induced temperature rise for a given SAR will depend on the RF frequency, which is dependent on the static magnetic field strength of the MR system. The RF-induced temperature rise for an RF array in MR systems of static magnetic field strengths of 7T is evaluated by suitable modification of the method described in ASTM F2182 standard.

Heating test was performed at three different positions (Fig. SC.1): Position 1) the array was immersed inside the gel phantom (rectangle: 15 cm × 20 cm, $\delta_{gel}$=0.5 S/m, $\varepsilon_{gel}$=77, heat capacity=4154 J/kg.°C) to be placed in a location with maximum expected SAR estimated from EM simulation (Fig. SC.1a). Position 2) the array was placed over the surface of the gel phantom, where we expected to have possible SAR hot spots (Fig. SC.1b). Position 3) The array was placed inside the coil and the phantom was set on top of the array (the array was not in direct contact with the gel) to mimic the real MR imaging set up (Fig. SC.1c). The array was covered with a thin layer of plastic to avoid the direct contact of the array with the gel material.

For each position, the thermal test was conducted in three different modes; i) array were decoupled from RF excitation using antiparallel diodes (for 4 elements located inside the coil), ii) coupled array (no antiparallel diodes), and iii) control experiment, which had no RF array. For each thermal experiment SAR was calculated as:

$SAR=C_{hc}\frac{dT}{dt}$ (15)

where *C_hc_* is the heat capacity, *T* is the temperature and *t* is the time.


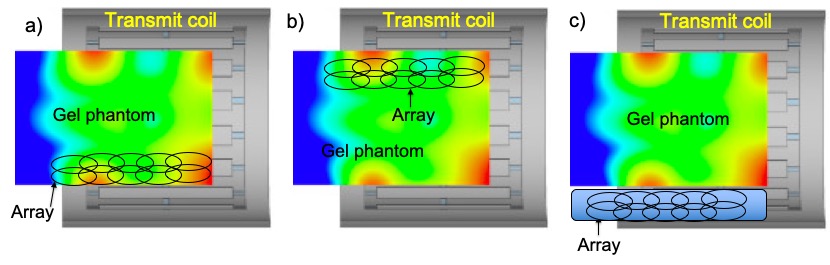


**Fig. SC.1**. a) Position 1: the array was immersed inside the gel phantom to be placed in a location with maximum expected SAR estimated from EM simulation. b) Position 2: The array was placed over the surface of the gel phantom with estimated possible high SAR hot spots. c) Position 3: The array was placed inside the coil and phantom was set on top of the array to mimic the real MR imaging set-up.

For all positions, the test procedure is divided into two steps. In Step 1, the temperature rise on or near the array at several locations is measured using fiber-optic thermometry probes during approximately 15 min of RF application. In Step 2, the array is removed and the same RF application is repeated while the temperature measurements are obtained at the same probe locations as in Step 1. In all measurements (with and without the array) the position of the fiber optic probes relative to the phantom was kept constant. The local SAR is calculated from the temperature measurements for each probe location. Figure SC.2 shows the experiment set-up.

We visually (by eye on the actual apparatus) examined the location of the probes relative to the RF array, immediately before and after the heating assessment because significant variations in the measured temperature can occur related to slight variability in probe positions relative to the array. Therefore, we used the exact same location of the probe when studying the temperature changes occurring with and without the array.


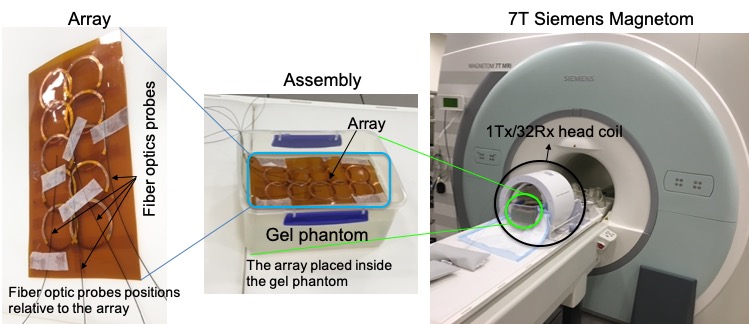


**Fig. SC.2**. Set-up for temperature measurements at 7T MRI using gel phantom. This figure does not reflect any specific mode explained in the text. This is just a general figure to show the experimental set-up.

*Heating test Results*

Position (1): Expected location of peak local SAR (worst case)

Table S1 summarizes all measured temperatures and calculated SARs for position 1.

Table S1: Summarized Temperature (Tem) and SAR results under position 1. The experiment was performed at three different modes. Four temperature probes were used at each mode to collect temperature at various location in the vicinity of the array.

| Mode/Tem | ***ΔT_p1_***  °C | ***ΔT_p2_***  °C | ***ΔT_p3_***  °C | ***ΔT_p4_***  °C | ***dT/dt_p1_*** x 10^-5^ (°C/s) | ***dT/dt_p2_*** x 10^-5^  (°C/s) | ***dT/dt_p3_*** x 10^-5^  (°C/s) | ***dT/dt_p4_*** x 10^-5^  (°C/s) | **SAR_p1_**  (W/kg) | **SAR_p2_**  (W/kg) | **SAR_p3_**  (W/kg) | **SAR_p4_**  (W/kg) |
| --- | --- | --- | --- | --- | --- | --- | --- | --- | --- | --- | --- | --- |
| **Tuned** | 0.71 | 0.63 | 0.61 | 0.64 | 75.13 | 66.67 | 64.55 | 67.72 | 3.18 | 2.77 | 2.68 | 2.81 |
| **Detuned** | 0.55 | 0.52 | 0.50 | 0.51 | 58.20 | 55.03 | 52.91 | 53.97 | 2.42 | 2.28 | 2.19 | 2.24 |
| **Control** | 0.50 | 0.48 | 0.46 | 0.48 | 52.91 | 50.79 | 48.68 | 50.79 | 2.19 | 2.11 | 2.02 | 2.11 |

After 15 min of RF transmission, a maximum temperature increases of 0.55°C and 0.71°C were experienced at the decoupled (detuned) and coupled (tuned) RF arrays, respectively. Corresponding SAR gains (array SAR divided by control SAR) of 1.11 for the detuned array and 1.45 for the tuned array were calculated relative to the counterpart point in control (without array) mode (Table S2).

Table S2: Peak local SAR values obtained from experimental high SAR sequence at three different modes under position 1.

|  | **SAR_max_ (W/kg)* | *SAR gain* | **ΔT_max_ (°C)* |
| --- | --- | --- | --- |
| Tuned | 3.18 | 1.45 | 0.71 |
| Detuned | 2.42 | 1.11 | 0.55 |
| Control | 2.19 | 1.00 | 0.50 |

** SAR_max_* and ***Δ****T_max_* are the maximum calculated SAR and maximum measured temperature rise, respectively.

Position (2): The array was place over the surface of the gel phantom, where the array was immersed a few mm inside the phantom.

Table S3 summarizes all measured temperatures and calculated SARs for the position number 2.

Table S3: Summarized Temperature (Tem) and SAR results under position 2. The experiment was performed at three different modes. Four temperature probes were used at each mode to collect temperature at various location in the vicinity of the array.

| Mode/Tem | ***ΔT_p1_***  °C | ***ΔT_p2_***  °C | ***ΔT_p3_***  °C | ***ΔT_p4_***  °C | ***dT/dt_p1_*** x 10^-5^ (°C/s) | ***dT/dt_p2_*** x 10^-5^  (°C/s) | ***dT/dt_p3_*** x 10^-5^  (°C/s) | ***dT/dt_p4_*** x 10^-5^  (°C/s) | **SAR_p1_**  (W/kg) | **SAR_p2_**  (W/kg) | **SAR_p3_**  (W/kg) | **SAR_p4_**  (W/kg) |
| --- | --- | --- | --- | --- | --- | --- | --- | --- | --- | --- | --- | --- |
| **Tuned** | 0.68 | 0.58 | 0.58 | 0.61 | 71.96 | 61.37 | 61.37 | 64.55 | 2.97 | 2.67 | 2.67 | 2.81 |
| **Detuned** | 0.53 | 0.49 | 0.46 | 0.49 | 56.08 | 51.85 | 48.42 | 51.85 | 2.33 | 2.15 | 2.19 | 2.15 |
| **Control** | 0.47 | 0.44 | 0.41 | 0.47 | 49.47 | 46.56 | 43.16 | 49.47 | 2.05 | 1.93 | 1.79 | 2.05 |

After 15 min of RF transmission, a maximum temperature increases of 0.53°C and 0.68°C were experienced at the decoupled (detuned) and coupled (tuned) RF arrays, respectively. Corresponding SAR gains (array SAR divided by control SAR) of 1.14 for the detuned array and 1.45 for the tuned array were calculated relative to the counterpart point in control (without array) mode (Table S4).

Table S4: Peak local SAR values obtained from experimental high SAR sequence at three different modes under position 2.

|  | **SAR_max_ (W/kg)* | *SAR gain* | **ΔT_max_ (°C)* |
| --- | --- | --- | --- |
| Tuned | 2.97 | 1.45 | 0.68 |
| Detuned | 2.33 | 1.14 | 0.53 |
| Control | 2.05 | 1.00 | 0.47 |

** SAR_max_* and ***Δ****T_max_* are the maximum calculated SAR and maximum measured temperature rise, respectively

Position (3): Approximate location of expected in vivo use, gel not in contact with the array.

Table S5 summarizes all measured temperatures and calculated SARs for the position number 3.

Table S5: Summarized Temperature (Tem) and SAR results under position 2. The experiment was performed at three different modes. Four temperature probes were used at each mode to collect temperature at various location in the vicinity of the array.

| Mode/Tem | ***ΔT_p1_***  °C | ***ΔT_p2_***  °C | ***ΔT_p3_***  °C | ***ΔT_p4_***  °C | ***dT/dt_p1_*** x 10^-5^ (°C/s) | ***dT/dt_p2_*** x 10^-5^  (°C/s) | ***dT/dt_p3_*** x 10^-5^  (°C/s) | ***dT/dt_p4_*** x 10^-5^  (°C/s) | **SAR_p1_**  (W/kg) | **SAR_p2_**  (W/kg) | **SAR_p3_**  (W/kg) | **SAR_p4_**  (W/kg) |
| --- | --- | --- | --- | --- | --- | --- | --- | --- | --- | --- | --- | --- |
| **Tuned** | 0.30 | 0.28 | 0.28 | 0.29 | 31.74 | 29.63 | 29.63 | 30.69 | 1.32 | 1.30 | 1.30 | 1.27 |
| **Detuned** | 0.27 | 0.27 | 0.25 | 0.26 | 28.57 | 28.57 | 26.45 | 27.51 | 1.19 | 1.19 | 1.19 | 1.14 |
| **Control** | 0.25 | 0.24 | 0.24 | 0.25 | 26.45 | 25.40 | 25.40 | 26.45 | 1.19 | 1.05 | 1.05 | 1.19 |

After 15 min of RF transmission, a maximum temperature increases of 0.27°C and 0.30°C were experienced at the decoupled (detuned) and coupled (tuned) RF arrays, respectively. Corresponding SAR gains (array SAR divided by control SAR) of 1.11 for the detuned array and 1.00 for the tuned array were calculated relative to the counterpart point in control (without array) set up (Table S6).

Table S6: Peak local SAR values obtained from experimental high SAR sequence at three different modes under position 3.

|  | **SAR_max_ (W/kg)* | *SAR gain* | **ΔT_max_ (°C)* |
| --- | --- | --- | --- |
| Tuned | 1.32 | 1.11 | 0.30 |
| Detuned | 1.19 | 1.00 | 0.27 |
| Control | 1.19 | 1.00 | 0.25 |

** SAR_max_* and ***Δ****T_max_* are the maximum calculated SAR and maximum measured temperature rise, respectively.

1. [↑](#footnote-ref-1)
